# Diastolic Mitral Regurgitation

**DOI:** 10.14797/mdcvj.1054

**Published:** 2021-12-15

**Authors:** Rowa Attar, K. Carlos El-Tallawi

**Affiliations:** 1 Houston Methodist DeBakey Heart & Vascular Center, Houston, Texas, US

**Keywords:** mitral regurgitation, echocardiography, Doppler, atrioventricular block

## Abstract

An 89-year-old female with a history of hypertension presented to the hospital with symptoms of fatigue. Her electrocardiogram (ECG) showed high-grade atrioventricular (AV) block, so a transthoracic echocardiogram was obtained to assess for structural heart abnormalities (Figure 1). Color Doppler showed mild mitral regurgitation (MR) extending into diastole. Temporal interrogation of the MR jet using continuous wave Doppler confirmed the diastolic component.

Diastolic MR is generally described in the setting of AV dissociation. In patients with high-degree AV block and underlying sinus rhythm, the prolonged diastolic time with accompanying superimposed left atrial (LA) contractions will lead to a significant elevation in left ventricular end-diastolic pressure (LVEDP), creating a reverse gradient favoring flow from the left ventricle back into the LA during diastole. Diastolic MR also can occur with substantial elevations in LVEDP in restrictive cardiomyopathies and acute severe aortic regurgitation.

**Figure 1:**
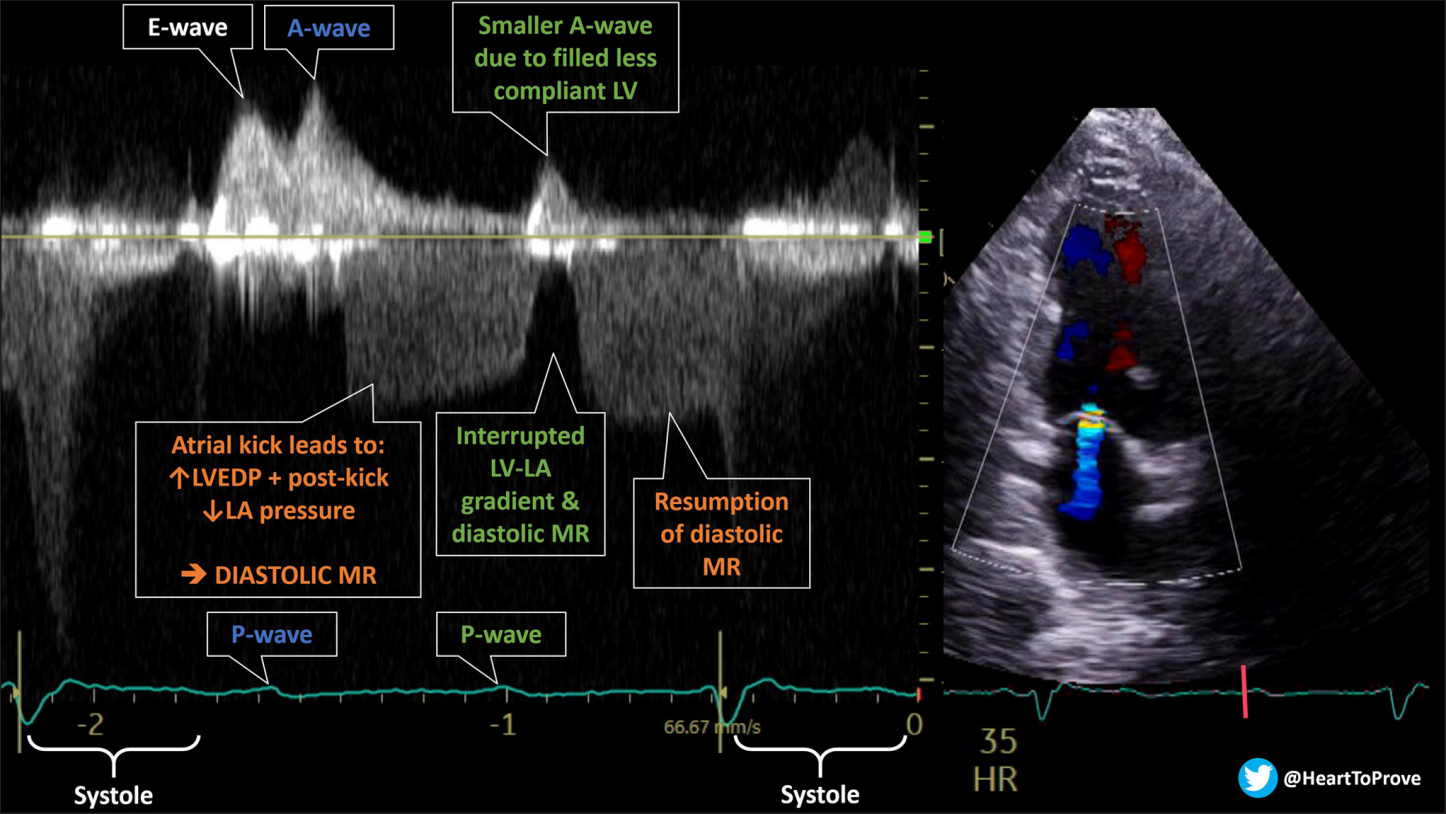
The hemodynamic elements of this mitral regurgitation (MR) are dissected and explained. MR: mitral regurgitation; LA: left atrial; LV: left ventricle; LVEDP: left ventricular end-diastolic pressure.

